# The Opening of the Medical Schools

**Published:** 1894-10-06

**Authors:** 


### The Hospital

AN INSTITUTIONAL JOURNAL OF

The Medical Sciences and Hospital Administration,

WITH

AN EXTRA NURSING SUPPLEMENT.

VOL. XVII.—No. 419.] [OCT. 6, 1894.

### THE OPENING OF THE MEDICAL SCHOOLS.

To the "freshman" in medicine the address at the opening of his first session is a very great occasion indeed. To his father, when he happens to be a medical man come up to see his son established at his own old school, it may be a mere tedium and weariness of the flesh. So, at any rate, the *Times* tells us, and it ought to know. In a languid article the leading journal discourses on what might be done by the orators who open the London medical session, but is not. Perhaps those orators might, if occasion served, have something to say about opportunities wasted or feebly used by leader writers in great journals. At any rate, it seems to us that a reader who brings some freshness of spirit to the task of reading the opening addresses of the present medical session will find a good deal of material well worthy of consideration and capable of stimulating the mind.

The opening addresses are delivered and addressed to students. The *Times* critic thinks they should be delivered to students but addressed to the public. The orators, wisely we think, do not take that view. The public does not want to hear a very great deal about medicine and medical study, the students do. A carefully thought out address by one who has had experience of medical life and work to a young man who is just entering upon that life, can hardly fail to fulfil the function at least of a candle in a room previously dark. It may, when the lecturer is of superior capacity, be as the sunrise and the break of day.

Perhaps there never was a time when a greater service could be rendered to students by opening addresses than at the present. Medicine is on fire with haste. Many even among experienced practitioners, in the multitude of new discoveries and new theories, hardly know where they stand. It is almost impossible but that the youthful student shall catch the contagion of the times, and amid so many stars and blazing comets of novelty and splendour shall be tempted to think that solid plodding work is out of date. Some of the addresses well fulfil the function of steadying the astonished youth, and pointing out to him the one and only pathway by which he can arrive surely at the goal he seeks. At St. George's Hospital, for example, Dr. Isambard Owen took an infinity of pains to clear the ground before the feet of the medical neophyte, and to present to him a finished picture of himself as a prudent, well-guided, honest, and successful student.

The word "scientific," Dr. Isambard Owen told his hearers, is a very much abused term, meaning in many mouths little more than the word "Mesopotamia" in the mouth of the old woman who ascribed her conversion to its utterance in melting tones. This surely is a remonstrance which not junior medical students only, nor even practitioners, but the whole public which reads and writes, may very profitably give heed to. "Students must beware," Dr. Owen said, "of being misled by a trivial sense occasionally attached to the words 'science' and 'scientific,' even by some professed authorities on medical education. There was a popular idea that 'scientific' meant something done with a test tube, or a microscope, or a scalpel, and that only the studies of the laboratory deserved the name. The problems of clinical work were even more complex than those of the laboratory, the training they required longer and severer." Now clinical work, the work of the bedside, is the true and essential business of the medical profession as a whole. It is well that physiological science, as that all science should progress; but inasmuch as the business of the doctor is to practice physic in the concrete, not to seek his own pleasure in the study of the problems of physiology in the abstract, it is plain that the ordinary medical student, and even the extraordinary one, whose intention it is to be a practising doctor, must study physiology and every other "pure science" as a means to an end. In other words, medical studies must be directed to the production of medical men. These are, of course, truisms to the aged and *blasé* medical transcendentalist; but to the youthful student whose hand is placed for the first time on the latch of the medical gate, they are as the breath of life, indispensable.

Some of the lecturers, notably Dr. Robert Boxall and Mr. Gustavus Hartridge, did address their lectures, at any rate to some extent, to the public. Mr. Hartridge told the public that it did not understand enough of science to estimate medical men at their true value. If it did it would make them members of the House of Commons, and even insist upon the elevation of some of them to the Peerage. It is quite certain that the medical training fits men in a remarkable measure for the patient and systematic study of problems of a practical nature; and this seems to us to be eminently the kind of training which the politician of the moment needs. The days of large and fundamental constitutional changes are past in British politics. The rhetoric of a Gladstone, the epigram of a Beaconsfield are no longer required. What is required is the systematic study of small and large problems of a practical order which affect the social condition of the people. No kind of training, it seems to us, is better fitted for the politician than the medical, which presents problems for solution of a variety and complexity only to be matched by those which are presented for daily solution by the social organism. The question touched by Mr. Hartridge of raising medical men to the peerage is a very old one. The fact that they are not raised to the peerage is quite striking, and is one of many proofs that intellect, however capacious and luminous it may be, is no match for established and unabashed privilege in the race for social precedence.

Dr. Boxall discussed the question of the use and abuse of hospitals—a singular subject to bring before first year's medical students. But no doubt his intention was to speak to the parents and the profession rather than to the young men. If he is correctly reported his arguments appear to point one way and his conclusions another. One thing in this connection seems pretty obvious, namely, that hospitals, though they do a great deal for the student and still more for the consultant and the specialist, do not render that continuous service to the general practitioner which they are capable of rendering and which they ought to render. It cannot be denied that there is failure somewhere when nineteen-twentieths of the working profession gain no substantial advantage from the splendid educational resources of our admirably-equipped hospitals.

One thing, practically all the opening addresses delivered at the different medical schools have in common. Each of them presents to the youthful medical postulant a subject which is of keen and vital interest to the whole profession of which he desires to become a member. Reading them all he will feel as if he had taken a plunge *in medias res*. He may critically ask himself how he has liked the plunge and whether those various subjects which excite such eager interest in medical men are of the kind that are likely to be permanently interesting to himself. If he feels that they are dull to his mind, or worse still, distasteful, now is the time for him to face the question boldly of the possibility of mistake in his choice of a calling. This very useful purpose, at any rate, the opening addresses serve; and so long as they continue to be so carefully prepared and of such practical bearings as those of the present year we cannot but hope that they will also continue to lend an element of soberness and dignity to an annual function which might otherwise degenerate into a slovenly formality destitute of impressiveness and honour.

